# Facile preparation of highly-dispersed cobalt-silicon mixed oxide nanosphere and its catalytic application in cyclohexane selective oxidation

**DOI:** 10.1186/1556-276X-6-586

**Published:** 2011-11-08

**Authors:** Qiaohong Zhang, Chen Chen, Min Wang, Jiaying Cai, Jie Xu, Chungu Xia

**Affiliations:** 1 State Key Laboratory for Oxo Synthesis and Selective Oxidation, Lanzhou Institute of Chemical Physics, Chinese Academy of Sciences, Lanzhou 730000, People's Republic of China; 2 State Key Laboratory of Catalysis, Dalian National Laboratory for Clean Energy, Dalian Institute of Chemical Physics, Chinese Academy of Sciences, 457 Zhongshan Road, Dalian 116023, People's Republic of China

## Abstract

Highly dispersed cobalt-silicon mixed oxide [Co-SiO_2_] nanosphere was successfully prepared with a modified reverse-phase microemulsion method. This material was characterized in detail by X-ray diffraction, transmission electron microscopy, Fourier transform infrared, ultraviolet-visible diffuse reflectance spectra, X-ray absorption spectroscopy near-edge structure, and N_2 _adsorption-desorption measurements. High valence state cobalt could be easily obtained without calcination, which is fascinating for the catalytic application for its strong oxidation ability. In the selective oxidation of cyclohexane, Co-SiO_2 _acted as an efficient catalyst, and good activity could be obtained under mild conditions.

## Introduction

The preparation of a highly dispersed nanosphere with the desired properties has been intensively pursued not only for the fundamental scientific interest of the nanomaterials, but also for their wide technological applications. Up to the present, different methods, such as the Stöber method, a layer-by-layer deposition, a sol-gel process, or a hydrothermal method, etc., have been developed to prepare a highly dispersed nanosphere [1-5]. Various monocomponent nanospheres including SiO_2_, Fe_2_O_3_, CuO, ZnS, or metal materials Au and Pt could be successfully obtained [4-8]. These materials showed good properties during utilization in gas sensors, biomedicine, electrochemistry, catalysis, etc. Furthermore, for the demand of the application, much effort has been devoted to prepare a bi- or multicomponent nanocomposite [9-14]. Among these materials, silica was often utilized as a carrier to disperse the active phase on its surface or in its matrix because silica can not only be easily obtained from several precursors, but also remains stable in most chemical and biological environments. What's more is that the rapid development of the modern nanotechnolgy has supplied flexible methods to modulate the morphology and structure of silica, which could be adopted for the preparation of the SiO_2_-based nanocomposite [15,16].

Cobalt oxide system or cobalt-silicon mixed oxide is a widely studied system in material domain, which could be used as catalyst for many reactions involving hydrogen transfer, such as methane reforming, oxidation of hydrocarbon, Fischer-Tropsch synthesis, and hydrogenation of aromatics [17-22]. For the bi-component cobalt-silicon mixed oxide, it was acknowledged in the recent studies that the preparation method could show an obvious effect on the type and dispersion of cobalt oxide species, and thus on the catalytic performance of the derived catalysts [23-25]. For the traditional two-step method, silica was firstly prepared as a support, and then, cobalt species were introduced through ion-exchange, impregnation, or grafting techniques. Compared with this method, one-step condensation method owns it's predominance in that it allows a better control of the textural properties of the silica matrix and a more effective dispersion of cobalt oxide in the matrix on a nanometric scale.

From a particle-preparation point of view, microemulsion method is such a good method to prepare a uniform-sized nanosphere [26-29]. The water nanodroplets present in the bulk oil phase serve as nanoreactors to control the size and the distribution of the nanoparticles. While for cobalt-silicon mixed oxide, it seems that the uniform particle size distribution remains a delicate task with the normal sol-gel method or microemulsion methods [30-34]. In our previous work, a modified reverse-phase microemulsion method was successfully adopted to prepare a highly dispersed SiO_2_-based nanocomposite [35,36]. Herein, a similar method was used to prepare cobalt-silicon mixed oxide materials, and the obtained material presents as a kind of highly dispersed, uniform-sized nanosphere. In the catalytic application, this novel nanosphere showed a good activity for the selective oxidation of cyclohexane to cyclohexanol and cyclohexanone.

## Experiment

### Material preparation

Tetraethyl orthosilicate [TEOS] (99%), cobaltous acetate [Co(OAc)_2_·4H_2_O] (99%), ethanol [C_2_H_5_OH] (99.5%), acetone [C_3_H_6_O] (99.5%), cyclohexane [C_6_H_12_] (99.5%), *n*-butyl alcohol [C_4_H_9_OH] (99.5%), and aqueous ammonia [NH_3_·H_2_O] (28%) were obtained from Tianjin Kermel Chemical Reagent Development Center, Tianjin, China. Poly (oxyethylene) nonylphenol ether [NP-7] (industrial grade) was purchased from Dalian Chemical Ctl., Dalian, China. Cobalt oxide [Co_3_O_4_] (98%) denoted as C-Co_3_O_4 _was purchased from Tianjin Institute of Jinke Fine Chemical, Tianjin, China.

Firstly, two kinds of solution (solutions A and B) were obtained, respectively. Solution A was composed of 15.05 g of NP-7, 35.05 g of cyclohexane, and 8.05 g of *n*-butyl alcohol. Solution B was obtained with the addition of 2.00 g of NH_3_·H_2_O (16%) to the cobalt acetate aqueous solution (0.13 g of Co(OAc)_2_·4H_2_O and 5.35 g of deionized H_2_O). Microemulsion was obtained with the blending of solutions A and B. After stirring for 15 min, to this microemulsion, 5.2 g of TEOS was added slowly under stirring. After stirring was continued for 12 h, 10 ml of acetone was added to destroy the microemulsion. It was then centrifugated, washed with hot ethanol for three times, and dried at 353 K for 12 h. This material was denoted as Co-SiO_2_.

### Characterization

The microstructure of the material was examined by transmission electron microscopy [TEM] on an FEI Tecnai G2 Spirit electron microscope (FEI Company, Hillsboro, OR, USA) at an accelerating voltage of 100 kV. The surface morphology was observed by scanning electron microscopy [SEM] on an FEI Quanta 200F microscope (FEI Company, Hillsboro, OR, USA). The X-ray powder diffraction [XRD] patterns were obtained using Rigaku D/Max 2500 powder diffraction system (Rigaku Corporation, Tokyo, Japan) with Cu K*α *radiation with a scanning rate of 5°/min. Fourier transform infrared [FT-IR] spectra were collected between 4,000 and 400 cm^-1 ^on a Bruker Tensor 27 FT-IR spectrometer (Bruker Corporation, Billerica, MA, USA) in KBr media. Ultraviolet-visible diffuse reflectance spectra [UV-Vis DRS] were collected over a wavelength range from 800 to 190 nm on a Shimadzu UV-2550 spectrophotometer (Shimadzu Corporation, Kyoto, Japan) equipped with a diffuse reflectance attachment. X-ray absorption spectroscopy [XAS] measurement was performed at room temperature on the XAS Station of the U7C beam line of the National Synchrotron Radiation Laboratory (NSRL, Hefei, China).

### Catalytic oxidation of cyclohexane

Catalytic reactions were performed in a 100-ml autoclave reactor with a Teflon insert inside in which 0.12 g of catalyst, 15.00 g of cyclohexane, and 0.12 g of tert-butyl hydroperoxide [TBHP] (initiator) were added. When the reaction stopped, the reaction mixture was diluted with 15.00 g of ethanol to dissolve the by-products. The reaction products were identified by Agilent 6890N GC/5973 MS detector and quantitated by Agilent 7890A GC (Agilent Technologies Inc., Santa Clara, CA, USA) equipped with an OV-1701 column (30 m × 0.25 mm × 0.3 μm) and by titration. The analysis procedure was the same with that in the literature [21,37]. After the decomposition of cyclohexylhydroperoxide [CHHP] to cyclohexanol by adding triphenylphosphine to the reaction mixture, cyclohexanone and cyclohexanol were determined by the internal standard method using methylbenzene as an internal standard. The concentration of CHHP was determined by iodometric titration, and the by-products acid and ester, by acid-base titration. All the mass balances are above 92%.

## Results and discussion

**Figure 1:**
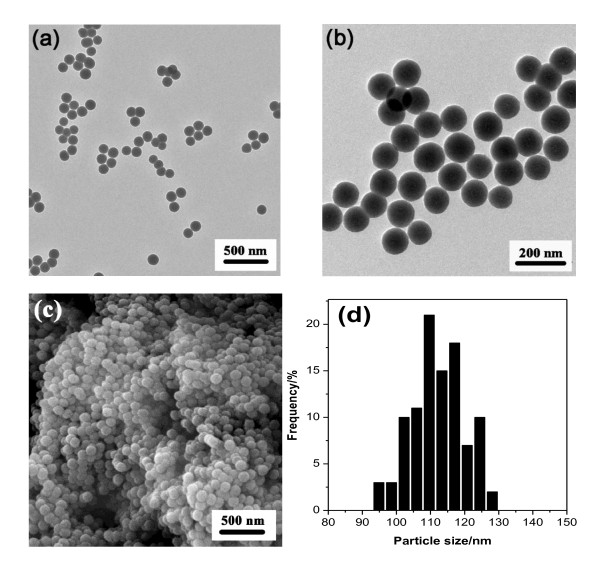
**TEM (a, b), SEM (c), and particle size distribution (d) of Co-SiO_2_**.

TEM and SEM were utilized to study the morphology of the material Co-SiO_2_. It can be observed in Figure 1a and 1b that the obtained material Co-SiO_2 _presented as a highly dispersed, uniform-sized nanosphere, which was further proved by the characterization of SEM (Figure 1c). The distribution of the particle size was centered at about 110 nm (Figure 1d). By comparison, in our previous work, the highly dispersed nanosphere could not be obtained with the normal operation of blending two microemulsions before adding a silicon source [38]. A similar situation also happened during the preparation of silica-supported cobalt materials [30,31]. As pointed out by Boutonnet et al., there are two main ways of preparing nanoparticles from the microemulsion method: (1) by mixing two microemulsions, one containing the precursor and the other, the precipitating agent; and (2) by adding the precipitating agent directly to the microemulsion containing the metal precursor [26]. Different with the above two methods, in the present work, the metal precursor was firstly prepared as an aqueous solution of a cobalt ammonia complex, which acted as the water phase in the microemulsion and could also supply a base environment for the hydrolysis of TEOS. No more bases are necessary to be added during the preparation process. This method can also avoid the blending of two microemulsions that might affect the properties of the water droplet in the microemulsion and then affect the morphology of the prepared materials. With the same method, highly dispersed Cu-SiO_2_, Ni-SiO_2_, and Zn-SiO_2 _nanospheres could also be successfully prepared.

**Figure 2:**
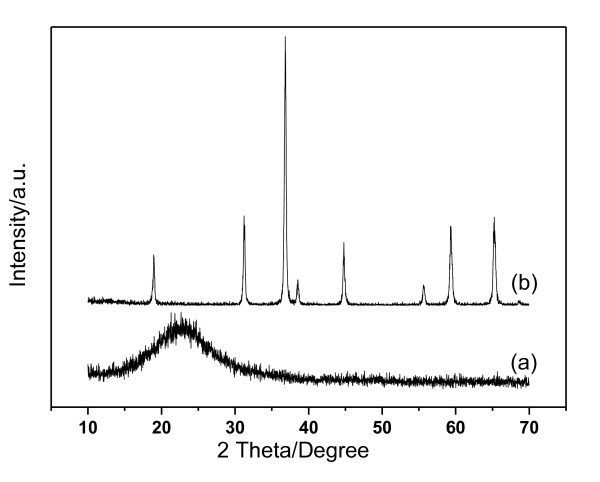
**XRD pattern of Co-SiO_2 _(a) and C-Co_3_O_4 _(b)**.

The composition of the material Co-SiO_2 _was primarily recognized through the XRD pattern measurement, which was shown in Figure 2. As a comparison, the pattern of the C-Co_3_O_4 _was also supplied in which eight peaks corresponding with the cubic structure of Co_3_O_4 _with the *Fd3m *space group can be clearly observed [21]. These peaks do not emerge in the pattern of Co-SiO_2_, and it shows only a broad peak at 2*θ *= ca. 22°, which is assigned to the amorphous silica. These results indicate that Co species in Co-SiO_2 _are amorphous and/or the particle size is too small [33].

**Figure 3:**
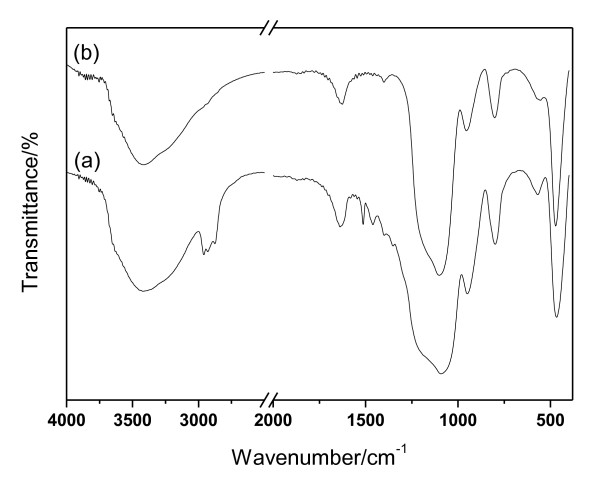
**FTIR spectra of the as-prepared sample (a) and Co-SiO_2 _(b)**.

The FTIR spectrum of the material Co-SiO_2 _is illustrated in Figure 3. Strong absorption bands at 1,090, 800, and 473 cm^-1 ^agree well with the SiO_2 _bond structure. The band at 1,090 cm^-1 ^was assigned to the asymmetric stretching vibration of the bond Si-O-Si in the SiO_4 _tetrahedron. The band at 800 cm^-1 ^was assigned to the vibration of the Si-O-Si symmetric stretching vibration. The band at 473 cm^-1 ^is related to the bending modes of the Si-O-Si bonds [37,39]. Besides these three bands, one weak shoulder band emerged at 960 cm^-1 ^that was usually attributed to the Si-OH stretching vibration. The absorption bands at 3,440 and 1,635 cm^-1 ^were caused by the absorbed water molecules [40]. For the as-prepared sample without solvent extraction, intense characteristic absorption bands associated with C-H bond (about 1,500 and 3,000 cm^-1^) are evident for the presence of the organic surfactant, which almost disappeared for the spectrum of Co-SiO_2_. This indicates that the surfactant could be totally removed with the solvent extraction method.

**Figure 4:**
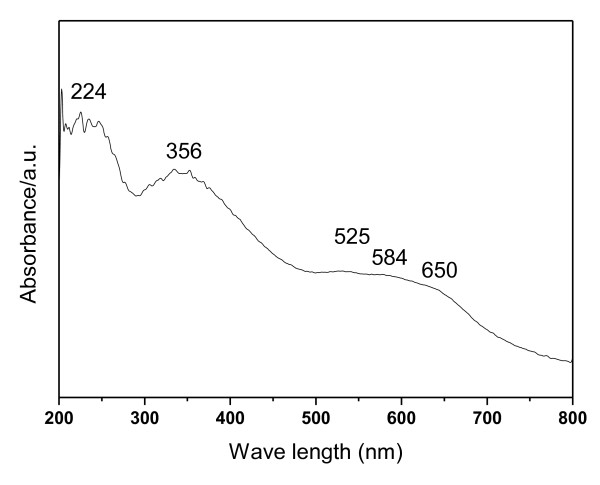
**UV-Vis DRS of Co-SiO_2_**.

UV-Vis DRS is a powerful characterization method to study the coordination geometry of cobalt incorporated in the materials, and the spectrum of Co-SiO_2 _was shown in Figure 4. Between 450 and 750 nm, this spectrum displays three absorption peaks (525, 584, and 650 nm), which can be unambiguously assigned to the ^4^A_2_(F) → ^4^T_1_(P) transition of Co(II) ions in tetrahedral environments [41,42]. Moreover, a broad band in the UV region centered at 224 nm is also observed. This has been assigned to a low-energy charge transfer between the oxygen ligands and central Co(II) ion in tetrahedral symmetry [43]. Besides the above absorption, another broad absorption was centered at 356 nm, which was assigned to Co(III) species [44]. It could be found in the literature that Co(III) was usually obtained through a heating treatment such as calcination [21,32,33]. In the present work, however, Co(II) salt precursor was firstly converted to cobalt(II) ammonia complex during the preparation process. The formation of a Co(II) ammonia complex would decrease the standard potential of Co(III)/Co(II) from 1.84 to 0.1 v, and then Co(III) ions were formed via the automatic oxidation of the Co(II) ammonia complex by dissolved dioxygen. As identified in a previous study [42], the emergence of this absorption was taken as a strong evidence for the presence of a distinct Co_3_O_4 _phase. So, it can be deduced from the above results that a Co_3_O_4 _phase exists in the material Co-SiO_2_.

**Figure 5:**
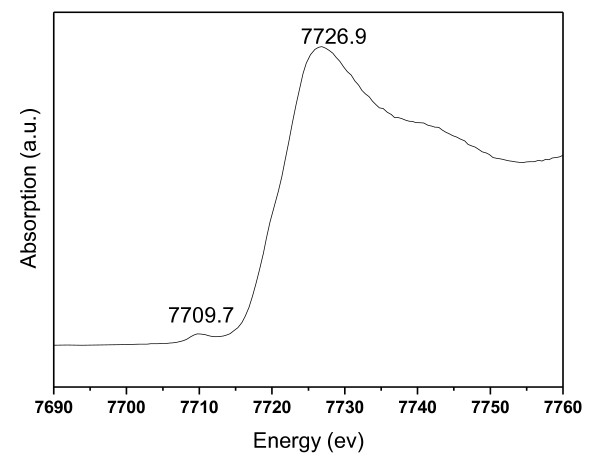
**XANES of Co-SiO_2_**.

In addition, from the characterization result of X-ray absorption spectroscopy near-edge structure [XANES] measurement (Figure 5), the information about the valence state of cobalt ions could be further acknowledged. It was believed that the main-edge should be shifted to a higher energy with the mixing of Co(III) with Co(II), and the distance between the pre-edge peak and the main edge can be used to measure the oxidation state of cobalt ions. Compared with the reference data, Co-SiO_2 _has an edge position that is consistent with cobalt ions aligning with Co_3_O_4 _that contains both oxidation states, not with CoO or CoAl_2_O_4 _[45]. The main-edge emerged at a higher energy (7,726.9 ev) for Co-SiO_2_, and the distance between the pre-edge peak and the main edge (*E*_main-edge _- *E*_pre-edge_) reached 17.2 ev. These situations are quite similar with those of Co_3_O_4_, manifesting that cobalt ions in Co-SiO_2 _own a close coordination environment with the cobalt ions in Co_3_O_4 _[45]. This is consistent with the result of UV-Vis DRS.

Selective oxidation of cyclohexane to cyclohexanone and cyclohexanol (the so-called K-A oil) is the centerpiece of the commercial production of Nylon. Although many attempts have been made to develop various catalytic systems for this reaction, it continues to be a challenge [46-48]. The present industrial process for cyclohexane oxidation is usually carried out above 423 K and 1 to approximately 2 MPa pressure without catalyst or with metal cobalt salt as homogeneous catalyst. For obtaining higher selectivity of K-A oil (about 80%), the conversion of cyclohexane is always controlled by about 4% [48]. It is one of the lowest efficient technologies that have been put into application among the present petrochemical domain. The main reason for the low yield of K-A oil is that it is easily overoxidized to the acids and further transformed to other by-products.

In the present work (Table 1), when Co-SiO_2 _was used as catalyst for the selective oxidation of cyclohexane, encouraging results were obtained. Under more mild conditions (388 K, which is 35 K lower than that of the industrial process), the conversion reached 6.0%, while the selectivity of K-A oil reached as high as 85.7% at the same time. As a comparison, the commercial C-Co_3_O_4 _could give a moderate activity with a conversion of 3.8% and a K-A oil selectivity of 78.4%. In addition, compared with the reported data, the predominance of the present Co-SiO_2 _is evident. Under the same conditions, when cobalt acetate was used, which was a homogeneous catalyst being widely used in the industrial process, the conversion was only 3.3% and the selectivity of K-A oil was also below 80% [19]. Moreover, the activity of Co-SiO_2 _is higher than that of the cobalt-containing mesoporous silica [Co-HMS] system (Table 1). Through N_2 _physical adsorption-desorption measurement, it could be acknowledged that the BET surface area of Co-SiO_2 _is 60 m^2^/g and average pore size is about 17 nm, respectively, which manifest that most of the pores come from the aggregation of the nanospheres. So, the accessible catalytic active sites of Co-SiO_2 _should exist all on the outerface of the nanospheres, which is contrary with the situation for the porous materials such as mesoporous silica or molecular sieves. For those porous materials, most of the catalytic active sites exist on the interface of the pore. Though the surface area of Co-SiO_2 _is much lower than that of Co-HMS (682 m^2^/g) [37], the absence of a long channel of inner pore may facilitate the fast diffusion of the substrate and the oxygenated products. Thus, the primary oxygenated products such as cyclohexanone and cyclohexanol are easily desorbed from the surface of the catalyst, which would decrease the chance for them to be overoxided. This might be the main reason for the evident enhancement of the selectivity for K-A oil. The deeper study of the relationship between the structure of the material and the activity is underway.

**Table 1:** Catalytic oxidation of cyclohexane over the catalysts

Catalysts	Conversion (mol%)	K-A oil (mol%)	**Products distribution (mol%) **^**a**^				
			A	K	CHHP	Acid	Ester
Co-SiO_2_	6.0	85.7	45.7	40.0	0.3	10.3	3.7
C-Co_3_O_4_	3.8	78.4	50.4	28.0	9.3	10.8	1.5
Co(OAc)_2 _^b^	3.3	78.2	43.2	35.0	4.3	15.0	2.5
Co-HMS ^b^	4.8	76.9	39.6	37.3	0.4	15.6	7.1

Reaction was carried out with 0.12 g of catalyst and 0.12 g of TBHP in 15 g of cyclohexane at 388 K for 6 h under 1.0 MPa O_2_.^a ^A, cyclohexanol; K, cyclohexanone; CHHP, cyclohexylhydroperoxide; Acid, mainly adipic acid; Ester, mainly dicyclohexyl adipate; K-A oil, A and K.^b ^Results from Chen et al. [19] under the same reaction conditions.

## Conclusions

With a modified reverse-phase microemulsion method, highly dispersed cobalt-silicon mixed oxide nanosphere was successfully prepared for the first time. The utilization of cobalt ammonia complex as metal source is favorable not only for controlling of the morphology, but also for obtaining a high valence state cobalt without calcination. These two factors are fascinating for the catalytic application, and Co-SiO_2 _was found to act as an efficient catalyst for the selective oxidation of cyclohexane. Considering that many kinds of metal ions can be converted to metal ammonia complex, we can extend this method to prepare such highly dispersed SiO_2_-based nanocomposite, which might show good application properties for its specific morphology and structure.

## Competing interests

The authors declare that they have no competing interests.

## Authors' contributions

JX and CX designed the experiment. QZ and CC carried out the experiment and drafted the manuscript. MW and JC participated in some of the characterizations and performed the data analysis. All authors read and approved the final manuscript.
